# A new species of the genus *Cristimenes* Ďuriš & Horká, 2017 (Decapoda, Caridea, Palaemonidae)

**DOI:** 10.3897/zookeys.852.34959

**Published:** 2019-06-05

**Authors:** Jin-Ho Park, Sammy De Grave, Won Kim

**Affiliations:** 1 School of Biological Sciences, Seoul National University, Seoul, 08826, Republic of Korea Seoul National University Seoul South Korea; 2 Oxford University Museum of Natural History, Oxford University, Parks Road, Oxford, OX1 3PW, UK Oxford University Oxford United Kingdom

**Keywords:** *Cristimenesbrucei* sp. nov., crinoid associate, Indo-West Pacific, Hong Kong, Korea, phylogeny

## Abstract

A new species of crinoid-associated shrimp, *Cristimenesbrucei***sp. nov.**, is described based on specimens from Korea, although the species also occurs in Hong Kong and is likely more widespread. The new species is morphologically very similar to *C.commensalis*, but can be distinguished by the reduced supraorbital tooth on the carapace. *Cristimenesbrucei***sp. nov.** is clearly recovered as a monophyletic species through COI barcode and molecular phylogenetic analyses based on four genetic markers (COI, 16S, H3, 18S).

## Introduction

The genus *Cristimenes* Ďuriš & Horká, 2017 is associated with echinoderms (Ďuriš and Horká 2017) and currently consists of three species: *C.cristimanus* (Bruce, 1965), *C.zanzibaricus* (Bruce, 1967) (both associated with echinoids), and *C.commensalis* (Borradaile, 1915), associated with crinoids. All three species are widespread across the tropical regions of the Indo-West Pacific, and morphologically easily distinguished from related genera by the unique carpo-propodal articulation of the second pereiopod (Borradaile 1915; Barnard 1958; Bruce 1965, 1967, 1982a, 1984, 1989, 1996; Hayashi and Honma 2004; Marin and Savinkin 2007; Ďuriš and Horká 2017).

*Cristimenescommensalis* differs from the other two species by its host affiliation and can also be easily distinguished by the morphology of the ambulatory dactyli (Bruce 1965, 1967, 1980; Ďuriš and Horká 2017). The species has been recorded from various host crinoid species across the Indo-West Pacific, after it was described from Murray Island, Torres Strait, Australia in 1915 (Barnard 1958; Bruce 1982b; Marin and Savinkin 2007). Bruce (1979) already remarked upon variation in the supraorbital tooth and the lateral carina of specimens from Hong Kong in comparison to Indonesian specimens (Bruce 1982a, 1983). Specimens matching this morphology were obtained from Korea, and based on a morphological comparison as well as a molecular analysis are herein reported as a new species.

## Materials and methods

Fieldwork for this study was carried out and organised in Korea (2012–2018), the Philippines (2014, 2018), and Vietnam (2016–2018) by Seoul National University (**SNU**), Korea Institute of Ocean Science and Technology (**KIOST**), the University of the Philippines Visayas (**UPV**), and theInstitute of Tropical Biology (**ITB**). Host crinoids were collected during scuba diving and associated shrimps separated. All shrimps and tissue of host crinoids were preserved in 80% ethanol. The type series is deposited in the Marine Arthropod Depository Bank of Korea, Seoul National University, Seoul, Korea (**MADBK**); National Institute of Biological Resources, Incheon, Korea (**NIBR**) and the Oxford University Museum of Natural History, Oxford, United Kingdom (**OUMNH.ZC**). Postorbital carapace length (pocl, in mm) is used as the standard size measurement.

Molecular phylogenetic analyses were performed to elucidate the phylogenetic position of the new species within *Cristimenes*. Four species of *Cristimenes* (*C.commensalis, C.cristimanus*, *C.zanzibaricus*, and the new species) and three crinoid-associated shrimps *Araiopontoniaodontorhyncha* Fujino & Miyake, 1970, *Laomenesamboinensis* (De Man, 1888), and *Unguicarispilipes* (Bruce & Zmarzly, 1983) were selected as the ingroup, with *Palaemonellapottsi* (Borradaile, 1915) as the outgroup. Total genomic DNA was isolated from fifth pleopod tissue or eggs using the QIAamp® DNA Micro Kit (QIAGEN, Hilden, Germany), according to the manufacturer’s instructions. Two mitochondrial DNA fragments (cytochrome c oxidase subunit I (COI) and 16S rRNA) and two nuclear DNA fragments (histone 3 (H3) and 18S rRNA (18S)) were amplified by polymerase chain reaction (PCR) with the primer pairs LCO1490/HCO2198 (Folmer et al. 1994), 16S-ar/16S-1472 (Crandall and Fitzpatrick 1996; Palumbi et al. 2002), H3F/H3R (Colgan et al. 1998), 18Sa2.0/18S9r (Whiting 2002), respectively. PCR protocols followed Horká et al. (2016), with PCR products sent to Macrogen Inc. (Seoul, Korea) for purification and Sanger sequencing. Geneious v11.1.5 (http://www.geneious.com) was used to manipulate and confirm the sequencing data from both DNA strands before data analysis. Newly obtained sequences and additional sequences from GenBank are listed in Table 1.

**Table 1.** Specimens used in the phylogenetic analysis, with collection location, GenBank accession numbers (COI, 16S, H3, and 18S), and source references. N/A - not available.

Taxa	Location	Voucher ID	GenBank accession numbers				Source
			COI	16S	H3	18S	
*Cristimenesbrucei* sp. nov.	Korea	MADBK 120532_012	MK688394	MK688410	MK688426	MK688442	Present study
	Korea	SNU KR JH537	MK688395	MK688411	MK688427	MK688443	Present study
	Korea	MADBK 120532_015	MK688396	MK688412	MK688428	MK688444	Present study
	Korea	MADBK 120532_015	MK688397	MK688413	MK688429	MK688445	Present study
* Cristimenes commensalis *	Philippines	SNU PH PI13	N/A	MK688414	MK688430	MK688446	Present study
	Philippines	SNU PH PI36	N/A	MK688415	MK688431	MK688447	Present study
	Philippines	SNU PH PI261	MK688398	N/A	N/A	N/A	Present study
	Philippines	SNU PH PI263	MK688399	N/A	N/A	N/A	Present study
	Philippines	SNU PH PI264	MK688400	MK688416	MK688432	MK688448	Present study
	Taiwan	UO Tw12-48B	KU064993	KU170697	KU065081	KU064912	Horká et al. 2016
* Cristimenes cristimanus *	Philippines	SNU PH PC49	MK688401	MK688417	MK688433	MK688449	Present study
	Vietnam	SNU VI VI109	MK688402	MK688418	MK688434	MK688450	Present study
	Vietnam	SNU VI VI110	MK688403	MK688419	MK688435	MK688451	Present study
	Vietnam	UO V08-34	KU064994	KU064838	KU065082	KU064913	Horká et al. 2016
* Cristimenes zanzibaricus *	Taiwan	UO Tw12-86	KU065011	KU170696	KU065096	KU064925	Horká et al. 2016
* Laomenes amboinensis *	Philippines	SNU PH12	MK688405	MK688420	MK688436	MK688452	Present study
	Philippines	SNU PH PH76	MK688404	MK688421	MK688437	MK688453	Present study
	Taiwan	UO Tw12-49	KU064979	KU064825	KU065063	KU064898	Horká et al. 2016
* Unguicaris pilipes *	Philippines	SNU PH PI57	MK688406	MK688422	MK688438	MK688454	Present study
	Philippines	SNU PH PI68	MK688407	MK688423	MK688439	MK688455	Present study
*Unguicaris* sp.	Taiwan	NTOU 6687-09	KU065020	KU064863	KU065108	KU064937	Horká et al. 2016
* Palaemonella pottsi *	Philippines	SNU PH PI56	MK688408	MK688424	MK688440	MK688456	Present study
	Philippines	SNU PH PI58	MK688409	MK688425	MK688441	MK688457	Present study

COI sequence divergence within and between species were calculated using the Neighbor-Joining method (Saitou and Nei 1987) and the Kimura 2-parameter (K2P) distance method (Kimura 1980) within the MEGA6 (Tamura et al. 2013). Multiple sequence alignment was performed using MAFFT v7 (Katoh and Standley 2013) under the default parameters and then checked by eye; phylogenetic trees for the combined dataset were constructed by maximum likelihood (ML) analysis and Bayesian Inference (BI) approaches. The best-fitting substitution model for COI (HKY+I+G), 16S (HKY+G), H3 (GTR) and 18S (GTR+I+G) was determined by jModelTest v2.1.10 (Darriba et al. 2012) according to the Akaike Information Criterion (AIC; Akaike 1974). The ML analysis was carried out using RAxML v8.2.4 (Stamatakis 2006) using the model GTRGAMMA for each partition with 1,000 bootstrap runs. The BI analysis was carried out using MrBayes v3.1.2 (Ronquist and Huelsenbeck 2003). The combined dataset was run for 10 million generations, sampling every 500 generations, with the first 50% trees discarded as burn-in. Phylogenetic trees were visualised in iTOL v4.0.3 (Letunic and Bork 2016).

## Systematics

### Infraorder Caridea Dana, 1852

#### Family Palaemonidae Rafinesque, 1815

##### Genus *Cristimenes* Ďuriš & Horká, 2017

###### Cristimenes brucei sp. nov.

Taxon classificationAnimaliaDecapodaPalaemonidae

http://zoobank.org/05D90862-52D5-4C2F-A5CF-5E4F3251A40E

Figures 1 , 2 , 3 , 4 , 5 , 6 , 7 , 8A–B , 9A–C , 10A–C

Periclimenes commensalis l: Bruce 1982a: 236–238, fig. 2.

####### Material examined.

***Holotype*.** KOREA – Jeju Special Self-Governing Province • 1 ov. ♀ (pocl 3.80 mm); Munseom Island; 33°13'37"N, 126°34'8"E; depth 20 m; 16 Oct. 2015; JH Park leg.; on *Anneissiajaponica* (Müller, 1841); NIBRIV0000841118; ***Paratypes.*** KOREA – Jeju Special Self-Governing Province • 3 ♀♀ (pocl 2.2, 1.86, 1.83 mm); Munseom Island; 33°13'37"N, 126°34'8"E; depth 20 m; 16 Oct. 2015; JH Park leg.; on *A.japonica*; MADBK 120532_006 • 1 ov. ♀ (pocl 3.65 mm); same data; 16 Oct. 2015; JH Park leg.; on *A.japonica*; OUMNH.ZC.2018-03-022 • 2 ♀♀, 1 ♂ (pocl 3.34, 3.1, 1.6 mm); same data; 17 Oct. 2015; JH Park leg.; on *A.japonica*; MADBK 120532_007 • 3 ♀♀, 1 ♂ (pocl 2.85, 2.2, 1.5, 1.98 mm); same data; 08 Jul. 2016; JH Park leg.; on *A.japonica*; NIBRIV0000841119 • 1 ♀ (pocl 1.67 mm); same data; 08 Aug.2016; JH Park leg.; on *A.japonica*; OUMNH.ZC.2018-03-023 • 1 ♀ (pocl 2.45 mm); same data; 31 Mar. 2018; JH Park leg.; on *A.japonica*; MADBK 120532_017.

####### Additional material.

KOREA – Dadohaesang National Park • 1 ♀ (pocl 2.7 mm); Geomundo Island; 34°3'35"N, 127°16'57"E; depth 20 m; 5 Jul. 2014; JH Park leg.; on *A.japonica*; MADBK 120532_002 – Gyeongsangbuk-do • 1 ♀, 3 ♂♂ (pocl 1.95, 2.0, 1.7, 1.6 mm); Pohang-si, Guryongpo; 36°0'25"N, 129°35'10"E; depth 15 m; 22 Sep. 2016; JH Park leg.; on *A.japonica*; MADBK 120532_015 – Jeju Special Self-Governing Province • 5 ♀♀, 1 ♂ (pocl 2.5, 2.3, 2.2, 1.7, 1.45, 2.0 mm); Beomseom Island; 33°13'7"N, 126°30'50"E; depth 20 m; 28 Feb. 2015; JH Park leg.; on *A.solaster* (Clark, 1907); MADBK 120532_003 • 8 ♀♀, 1 ♂ (pocl 2.77, 2.6, 2.6, 2.5, 2.5, 2.4, 2.3, 2.1, 1.9 mm); same data; 16 May 2015; JH Park leg.; on *Catoptometrarubroflava* (Clark, 1908); MADBK 120532_004 • 1 ov. ♀, 6 ♀♀, 3 ♂♂ (pocl 3.43, 3.48, 3.21, 3.1, 3.08, 2.37, 2.3, 2.89, 2.54, 1.97 mm); same data; 17 May 2015; JH Park leg.; on *A.japonica*; MADBK 120532_005 • 1 ♀, 1 ♂ (pocl 1.83, 1.83 mm); Saeseom Island; 33°14'2"N, 126°33'49"E; depth 20 m; 30 Jan. 2016; JH Park leg.; on *A.japonica*; MADBK 120532_011 • 3 ♀♀, 2 ♂♂ (pocl 2.45, 2.24, 1.2, 2.0, 1.68 mm); Seopseom Island; 33°13'55"N, 126°35'51"E; depth 15 m; 28 Jan. 2016; JH Park leg.; on *A.japonica*; MADBK 120532_010 • 1 ♀, 2 ♂♂ (pocl 3.0, 2.16, 1.89 mm); same data; 28 Apr. 2016; JH Park leg.; on *A.japonica*; MADBK 120532_013 • 1 ♂ (pocl 2.27 mm); same data; 28 Apr. 2016; JH Park leg.; on *A.japonica*; OUMNH.ZC.2018-03-024 • 1 ♀ (pocl 3.5 mm); same data; 28 Apr. 2016; JH Park leg.; on *A.japonica*; OUMNH.ZC.2018-03-025 • 1 ♂ (pocl 1.89 mm); same data; depth 30 m; 16 Jul. 2015; JH Park leg.; on *A.solaster*; MADBK 120532_008 • 2 ♀♀, 2 ♂♂ (pocl 2.74, 1.05, 1.53, 1.47 mm); same data; depth 27 m; 31 Jan. 2016; JH Park leg.; on *A.japonica*; MADBK 120532_012 • 1 ♂ (pocl 1.9 mm); Jeongbang Waterfall point; 33°14'36"N, 126°34'16"E; depth 15 m; 18 Jul. 2015; JH Park leg.; on A.solaster; MADBK 120532_009 • 2 ♀♀, 2 ♂♂ (pocl 3.5, 2.76, 2.55, 2.0 mm); Jigwido Island; 33°13'36"N, 126°39'12"E; depth 20 m; 14 Apr. 2013; JH Park leg.; on *A.japonica*; MADBK 120532_001 • 1 ♂ (pocl 1.77 mm); Unjin Port point; 33°13'2"N, 126°14'42"E, depth 18 m; 20 Oct. 2016; SH Lee leg.; on *A.japonica*; MADBK 120532_016.

**Figure 1.**
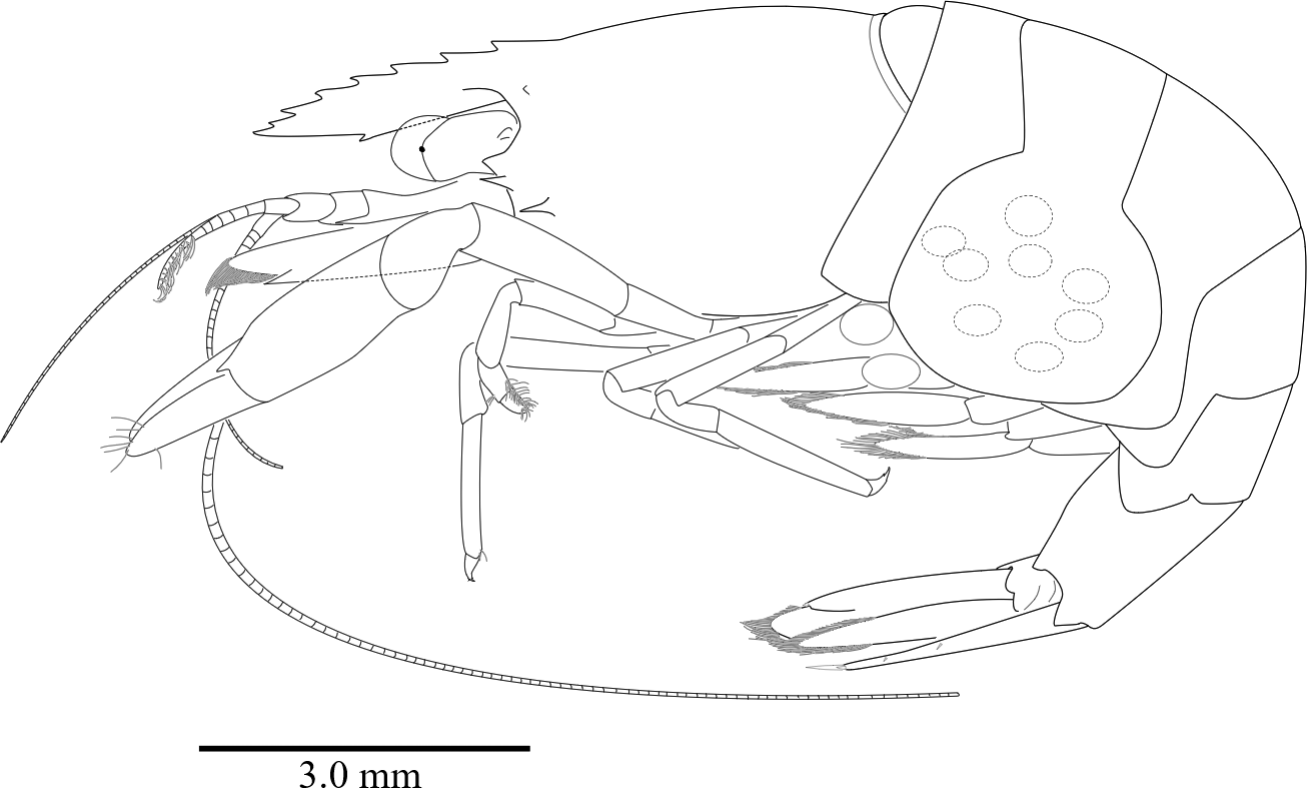
*Cristimenesbrucei* sp. nov., lateral aspect. Ovigerous female holotype pocl 3.65 mm (NIBRIV0000841118).

####### Diagnosis.

Rostrum well developed, with dorsal and ventral teeth. Carapace smooth, without epigastric tooth; lateral carinae feebly developed; supraorbital tooth reduced, blunt; inferior orbital angle pointed; antennal and hepatic teeth well developed. Fourth thoracic sternite without median process. Abdomen with rounded pleura. Telson with two pairs of small dorsal spiniform setae, and with three pairs of posterior spiniform setae. Eyes with hemispherical cornea. Basal antennular segment with two acute distolateral teeth. Antennal basicerite with sharp distoventral tooth; scaphocerite with large distolateral tooth, not reaching distal end of lamella. Epistome rounded. Mandible without palp; molar process robust; incisor process with four or five terminal teeth. Maxillula with bilobed palp. Maxilla with blunt palp, basal endite well developed, bilobed. First maxilliped with simple palp; basal and coxal endites fused; exopod with developed caridean lobe; epipod bilobed. Second maxilliped with subquadrate epipod, without podobranch. Third maxilliped with slender exopod; arthrobranch rudimentary. First pereiopods slender, fingers subspatulate with entire cutting edges. Second pereiopods equal in shape and subequal in size; palm articulated subproximally; cutting edges of fingers feebly dentate proximally, serrated distally. Dactyli of ambulatory pereiopods biunguiculate; corpus with two or three acute dorsodistal spinules, with acute preterminal accessory tooth. Uropodal exopod with distolateral tooth and movable acute spine.

####### Description.

**Figure 2.**
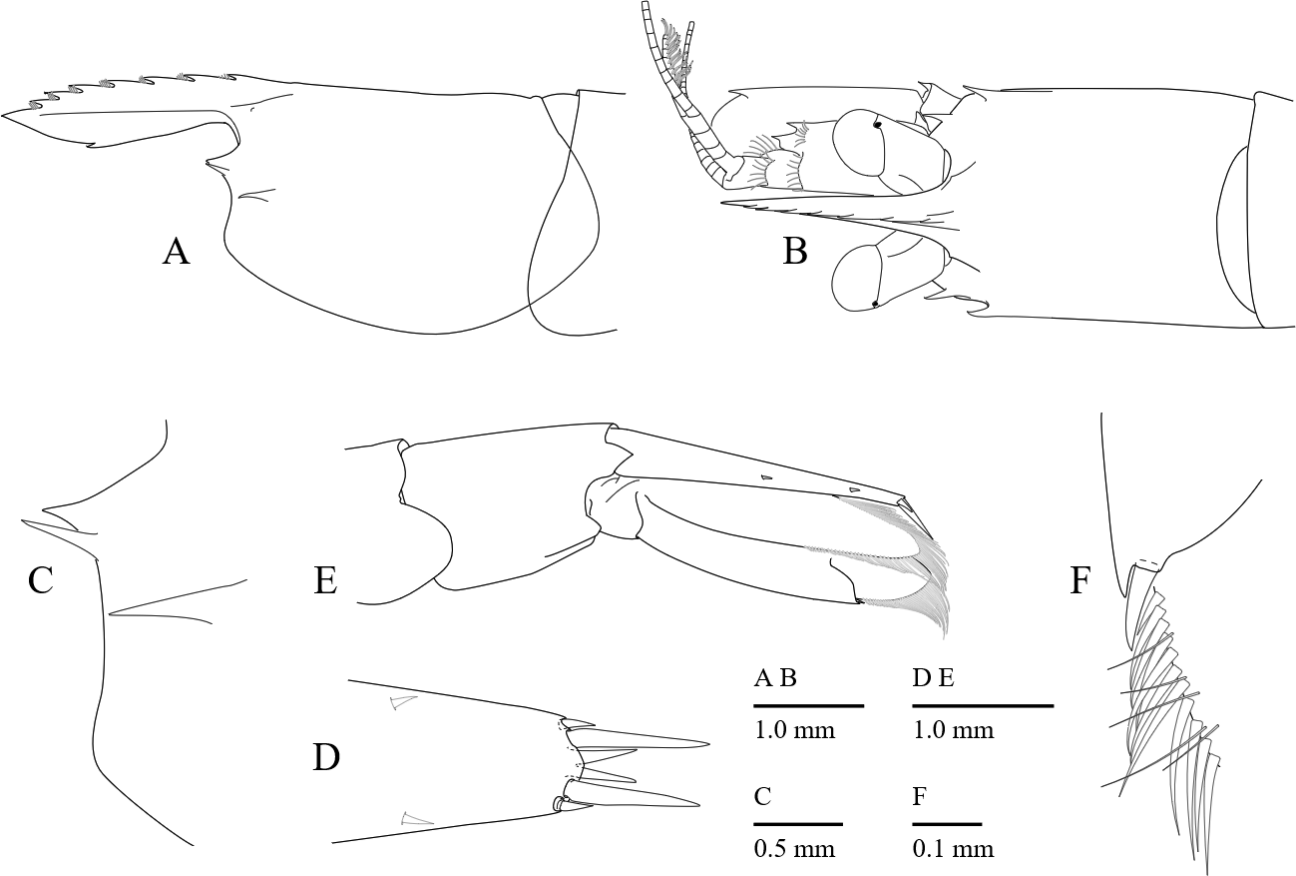
*Cristimenesbrucei* sp. nov., female pocl 2.74 mm (A, B, C, E) (MADBK 120532_012), ovig. female paratype pocl 3.65 mm (D) (OUMNH.ZC.2018-03-022). **A** carapace, lateral view **B** carapace, eyes, right antennule and antenna, dorsal view **C** anterior carapace, lateral view **D** distal end of telson, dorsal view **E** fifth and sixth abdominal segments, telson, and uropod, lateral view **F** left uropodal exopod, distolateral armature.

Rostrum (Figs 2A, B, 9A, B) well developed, slightly overreaching distal end of antennular peduncle; upper margin slightly convex with 6–8 dorsal teeth, ventral margin convex with 0–3 ventral teeth.

Carapace (Figs 2A, B, 9A, B) smooth without epigastric tooth; lateral carinae feebly developed; supraorbital tooth reduced, blunt (Fig. 9C); inferior orbital angle pointed; antennal and hepatic teeth (Fig. 2C) well developed, antennal tooth long and slender, hepatic tooth larger than antennal tooth; pterygostomial angle rounded.

**Figure 3.**
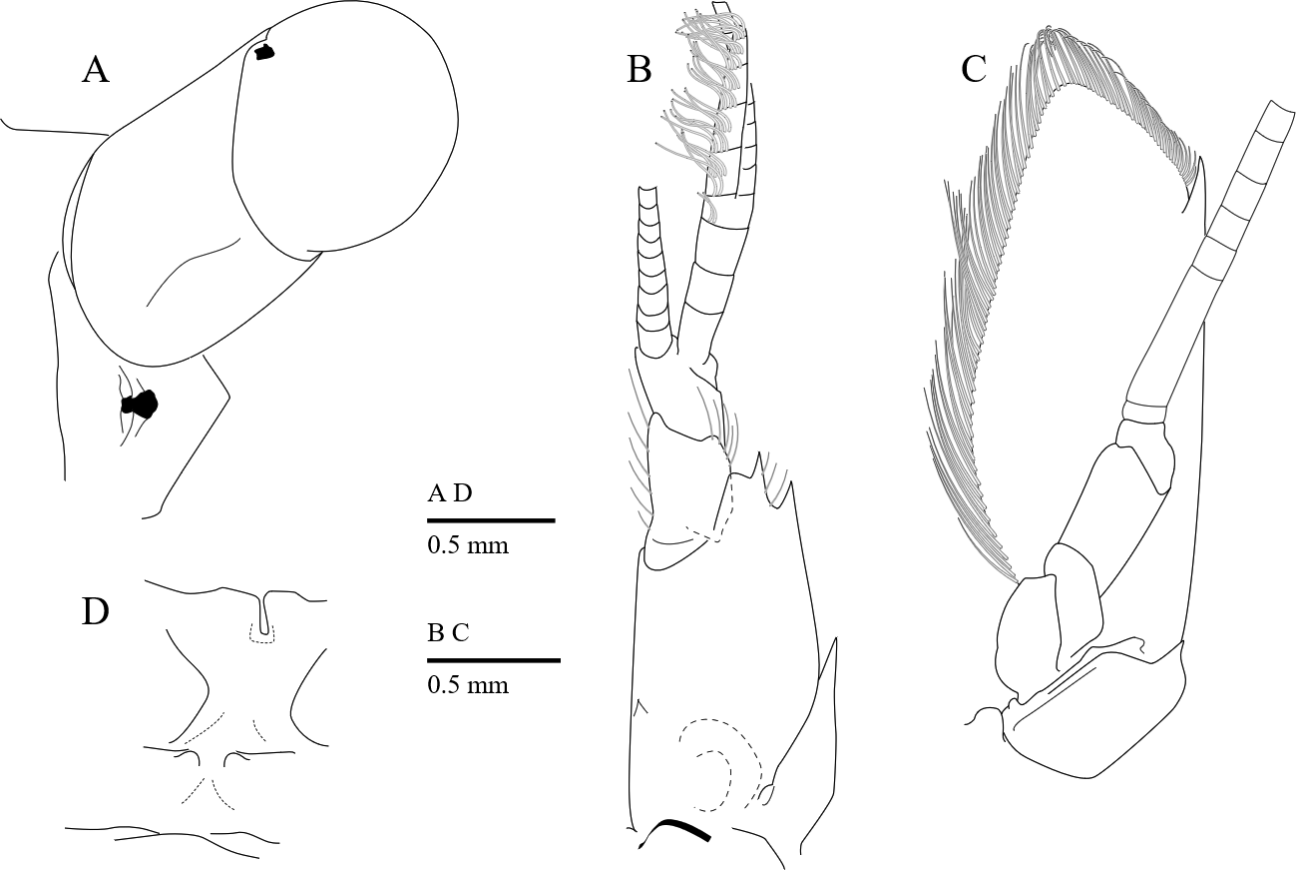
*Cristimenesbrucei* sp. nov., female pocl 2.74 mm (MADBK 120532_012). **A** eye, interocular region, and epistome, dorsal view **B** antennule, ventral view **C** antenna, ventral view **D** fifth (top) and fourth thoracic sternite.

Thoracic sternite (Fig. 3D) without special features; fourth thoracic sternite without finger-like median process.

Abdomen (Fig. 1) smooth; pleura of first to fifth segments rounded; sixth segment with pointed posterolateral angle, posteroventral angle blunt (Fig. 2E).

Telson (Fig. 2D, E) 0.78 of pocl, 3.2 times longer than proximal width; two pairs of small dorsal spiniform setae at 0.53 and 0.82 of telson length, with three pairs of posterior spiniform setae, outer pair short, inner pair long and stout.

Eye (Figs 2B, 3A) with hemispherical cornea, dorsolaterally with nebenauge; eyestalk 1.2 times as long as wide.

Antennule (Fig. 3B) well developed; basal segment with two acute distolateral teeth, with submarginal medioventral tooth; stylocerite reaching to middle of proximal segment; intermediate and distal segment subequal in length; upper flagellum biramous, proximal four segments fused, shorter free ramus with five segments, 0.3 of longer free ramus.

Antenna (Fig. 3C) well developed; basicerite with sharp distoventral tooth; ischiocerite and merocerite unarmed; carpocerite reaching to 0.4 of scaphocerite; scaphocerite 2.4 times as long as maximal wide, distolateral tooth large, not reaching distal end of lamellae.

**Figure 4.**
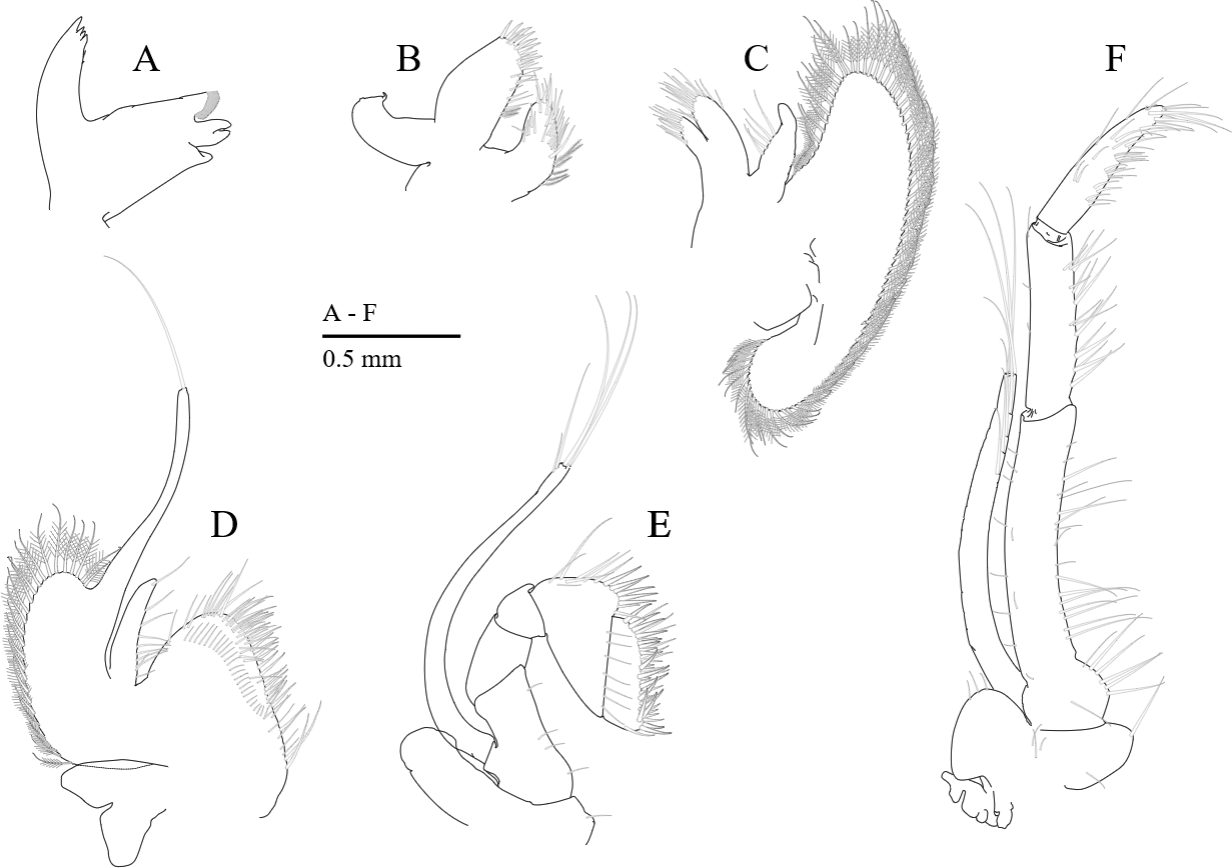
*Cristimenesbrucei* sp. nov., female pocl 3.48 mm (MADBK 120532_005). **A** mandible **B** maxillule **C** maxilla **D** first maxilliped **E** second maxilliped **F** third maxilliped.

Mandible (Fig. 4A) without palp; molar process robust, with four strong teeth and brush-like setae; incisor process with four or five terminal teeth.

Maxillula (Fig. 4B) with bilobed palp; upper lacinia broad, with stout and simple spines, with plumose setae on lower margin; lower lacinia robust with long spines distally, with plumose setae on lower margin.

Maxilla (Fig. 4C) with blunt palp, with sparsely plumose setae; coxal endite obsolete; basal endite well developed, bilobed, with sparsely plumose setae; scaphognathite 2.9 times as long as wide.

First maxilliped (Fig. 4D) with long simple palp, with sparsely plumose setae along the medial margin of the palp; basal and coxal endites fused, with serrulate setae medially; exopod with developed caridean lobe, flagellum with long simple seta; epipod bilobed.

Second maxilliped (Fig. 4E) with subquadrate epipod, without podobranch; merus and carpus without special features; propodus with slender simple setae; dactylus 2.7 times as long as wide, with dense serrulate setae distally.

Third maxilliped (Fig. 4F) with endopod slightly overreaching distal end of carpocerite; ischiomerus approximately six times longer than wide, medially sparsely setose; penultimate segment 0.56 length of ischiomerus, medially with long serrulate setae; terminal segment tapering, slightly downcurved distally, subequal to penultimate segment, with transverse rows of setae and group of terminal hamate setae; exopod slender with plumose setae distally; coxa with large rounded epipod, arthrobranch rudimentary.

**Figure 5.**
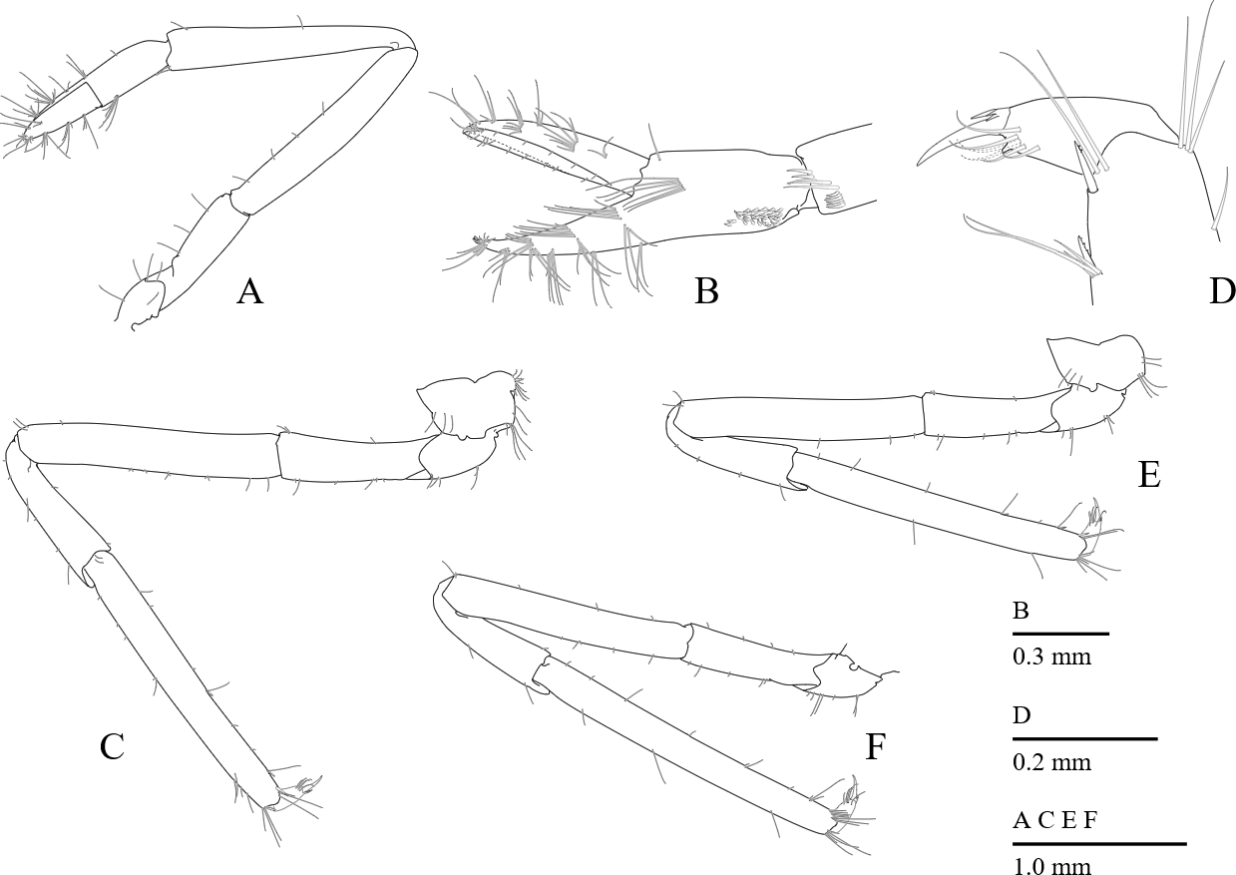
*Cristimenesbrucei* sp. nov., female pocl 2.74 mm (MADBK 120532_012). **A** first pereiopod, ventrolateral view **B** same, chela, lateral view **C** third pereiopod, lateral view **D** same, dactylus and distal propodus, lateral view **E** fourth pereiopod, lateral view **F** fifth pereiopod, lateral view.

First pereiopod (Fig. 5A, B) overreaching distal end of scaphocerite; ischium 0.56 length of merus, unarmed; merus and carpus subequal in length; carpus 1.36 times length of chela with row of serrulate setae along distomesial margin; chela 1.9 times longer than deep; palm with transverse row of serrulate setae ventrolaterally; fingers subspatulate, 0.89 times length of palm, cutting edge straight, entire, with groups of setae.

**Figure 6.**
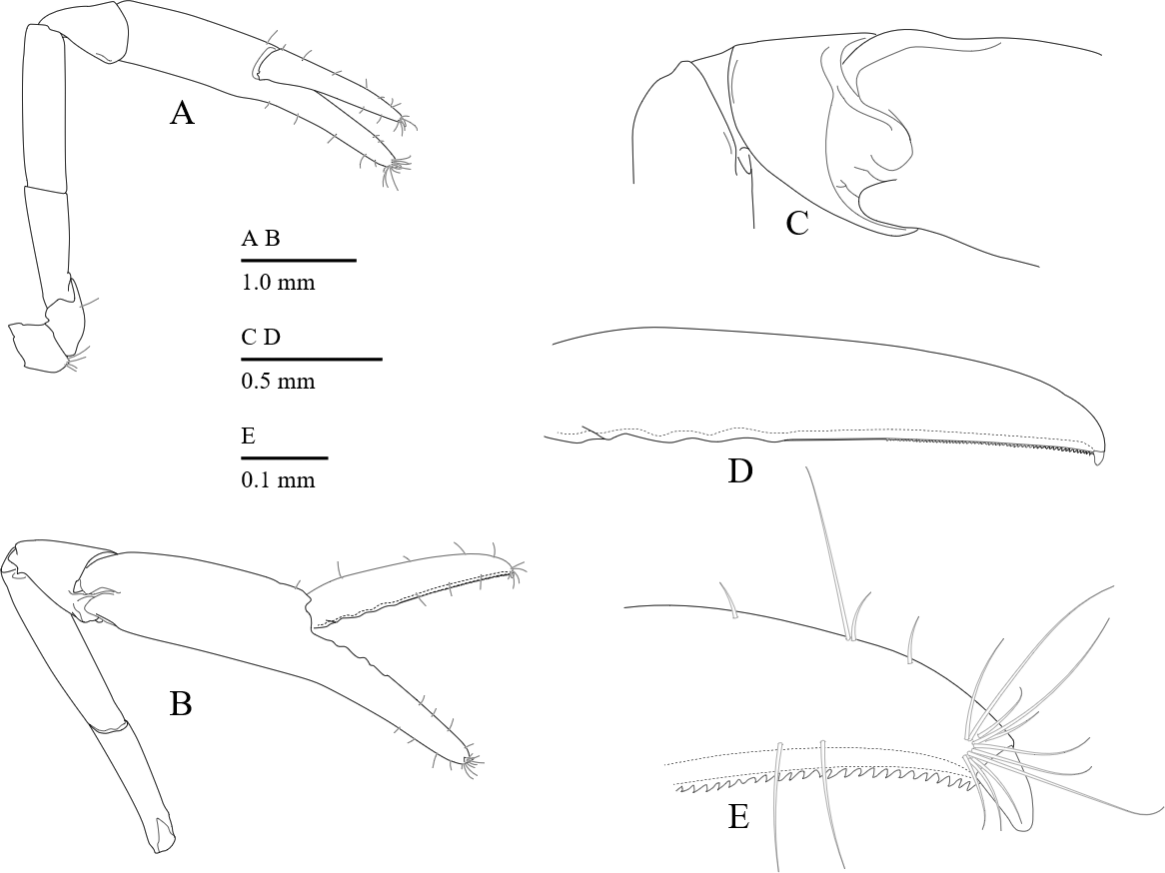
*Cristimenesbrucei* sp. nov., female pocl 2.74 mm (MADBK 120532_012). **A** minor right second pereiopod, ventrolateral view **B** major left second pereiopod, lateral view **C** same, carpo-propodal articulation, dorsal view **D** same, dactylus, lateral view **E** same, distal margin of cutting edge of dactylus.

Second pereiopods (Figs 6A, B, 10A) equal in shape and subequal in size; ischium 0.7 length of merus, unarmed; merus 2.0 times as long as carpus, unarmed; carpus short, 1.2 times as long as maximal width, articulated subterminally (Fig. 6C); palm cylindrical, 1.1 times as long as finger, articulated subproximally; fingers stout with curved tip; cutting edges of fingers feebly dentate proximally, serrated distally (Figs 6D, E, 10C, D).

Ambulatory pereiopods (Fig. 5C, E, F) of usual shape for genus, third pereiopod overreaching end of scaphocerite by distal margin of carpus. Third pereiopod (Fig. 5C) with ischium 0.54 length of merus, unarmed; merus 0.85 times length of propodus, unarmed; carpus 0.46 times length of propodus, unarmed; propodus with three distolateral spiniform setae including single distoventral one; dactylus 0.18 times length of propodus, biunguiculate; corpus with three acute dorsodistal spinules, with acute preterminal accessory tooth, ventral margin straight, with simple distal setae laterally; unguis 0.58 times as long as corpus (Fig. 5D).

Fourth pereiopod (Fig. 5E) with ischium 0.57 length of merus, unarmed; merus 0.80 times length of propodus, unarmed; carpus 0.42 times length of propodus, unarmed; propodus with four distolateral spiniform setae including two distoventral ones; dactylus 0.18 times length of propodus, biunguiculate; corpus with two acute dorsodistal spinules, with acute preterminal accessory tooth, ventral margin straight, with simple distal setae laterally; unguis 0.64 times as long as corpus.

Fifth pereiopod (Fig. 5F) with ischium 0.54 length of merus, unarmed; merus 0.74 times length of propodus, unarmed; carpus 0.38 times length of propodus, unarmed; propodus with five mesial spiniform setae, distolateral one absent; dactylus 0.18 times length of propodus, biunguiculate; corpus with two acute dorsodistal spinules, with acute preterminal accessory tooth, ventral margin straight, with simple distal setae laterally; unguis 0.70 times as long as corpus.

**Figure 7.**
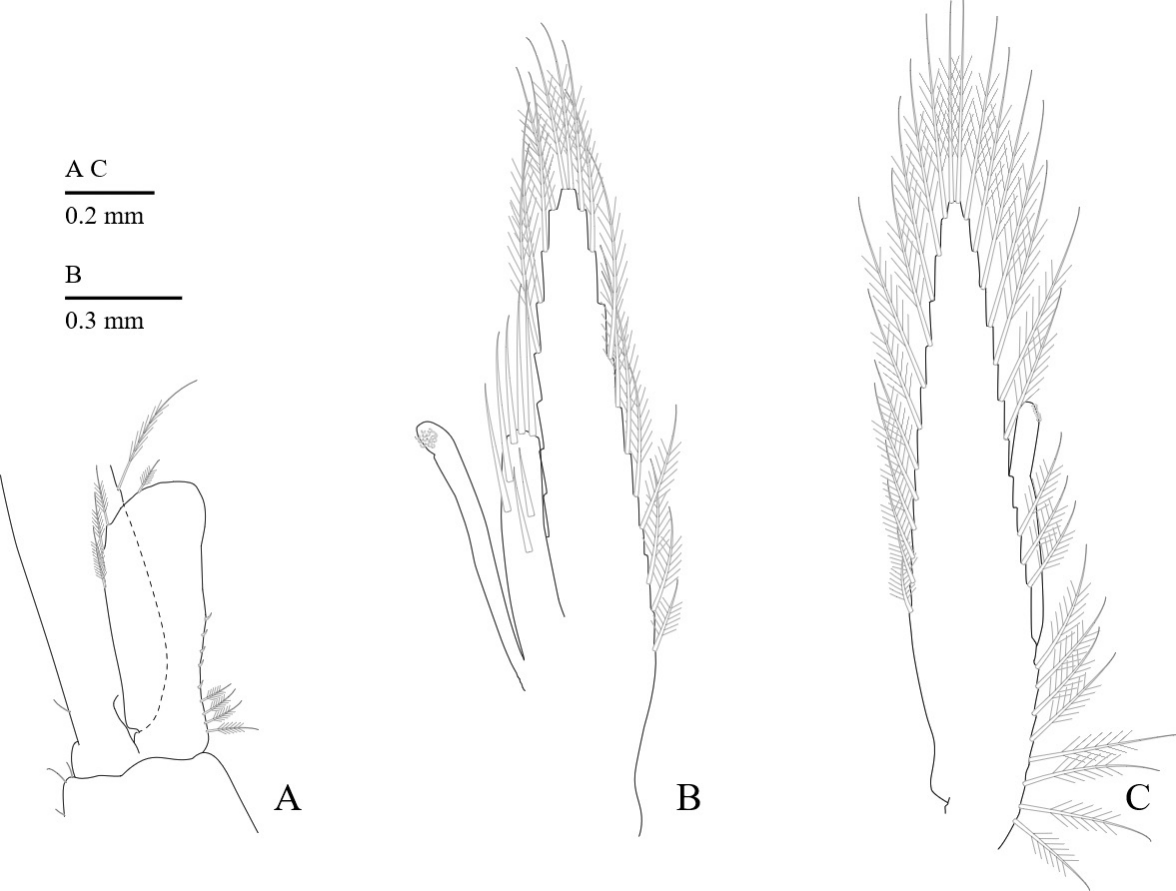
*Cristimenesbrucei* sp. nov., male pocl 2.54 mm (**A, B**) (MADBK 120532_005), female pocl 2.74 mm (**C**) (MADBK 120532_012) **A** endopod and basal half exopod of first pleopod **B, C** endopod of second pleopod.

Pleopods as usual for genus. First pleopod of male (Fig. 7A) with endopod 2.8 times longer than wide. Second pleopod of male (Fig. 7B) with appendix masculina with stout, long setae; appendix interna slightly longer than appendix masculina. Second pleopod of female (Fig. 7C) as usual for genus.

Uropod (Fig. 2E) overreaching distal end of telson; exopod with distolateral tooth and movable acute spine.

####### Etymology.

The new species is named in honour of Dr AJ (Sandy) Bruce, in recognition of his considerable contribution to the systematics of Palaemonidae.

####### Colour.

**Figure 8.**
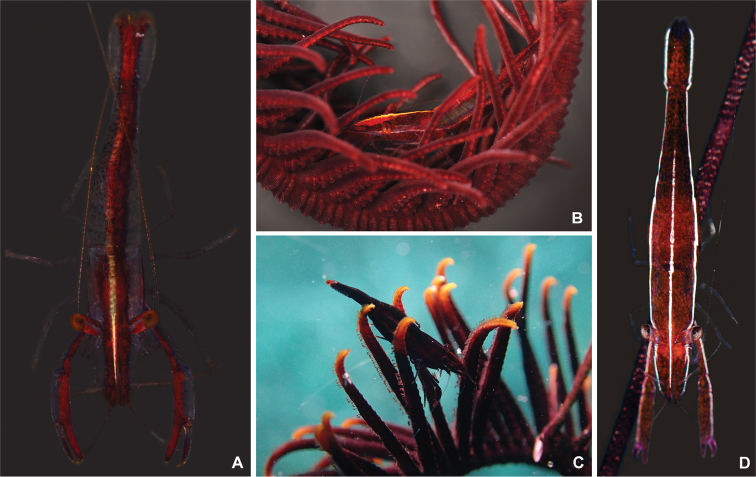
Colour pattern of three species of *Cristimenes*. **A***Cristimenesbrucei* sp. nov. from Korea (MADBK 120532_017) **B** same, with host crinoid species **C***Cristimenescommensalis* (Borradaile, 1915) from Vietnam (SNU VI VI305) **D***Cristimenescristimanus* (Bruce, 1965) from Vietnam (SNU VI VI297).

Body colour (Fig. 8A, B) orange or reddish-brown adapted to the colour of the host crinoids; creamy white line extending from the tip of the rostrum to the posterior dorsal margin of the carapace; similar, but thinner and lighter line extending from posterior ventral angle of the sixth abdominal segment to the lateral side of the first antennular peduncle.

####### Ecology.

The specimens were collected from the crinoids *Anneissiajaponica*, *A.solaster* and *Catoptometrarubroflava* at a depth of 15 – 27 m. Bruce (1982a) reported that the Hong Kong specimens were collected from *Tropiometraafra* (Hartlaub, 1890).

####### Distribution.

Presently only known from the type locality, Jeju Special Self-Governing Province, Korea as well as Hong Kong (Bruce 1982a).

####### Remarks.

**Figure 9.**
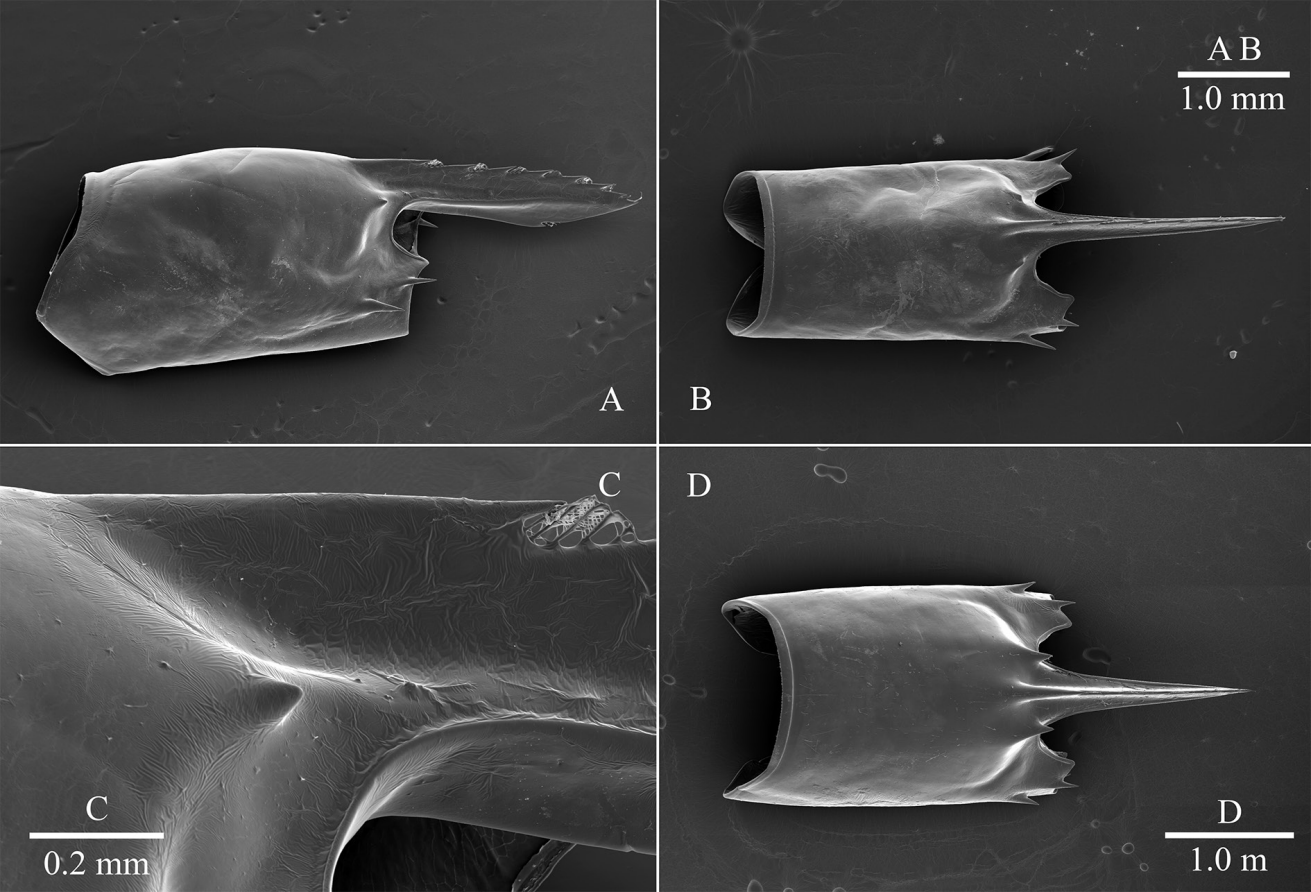
*Cristimenesbrucei* sp. nov., female pocl 2.7 mm (MADBK 120532_002) (**A, C**), male pocl 2.3 mm (MADBK 120532_005) (**B**), *Cristimenescommensalis* from Vietnam, female pocl 2.1 mm (SNU VI_VI229) (**D**) **A** carapace, lateral view **B** carapace, dorsal view **C** supraorbital tooth, lateral view **D** carapace, dorsal view.

**Figure 10.**
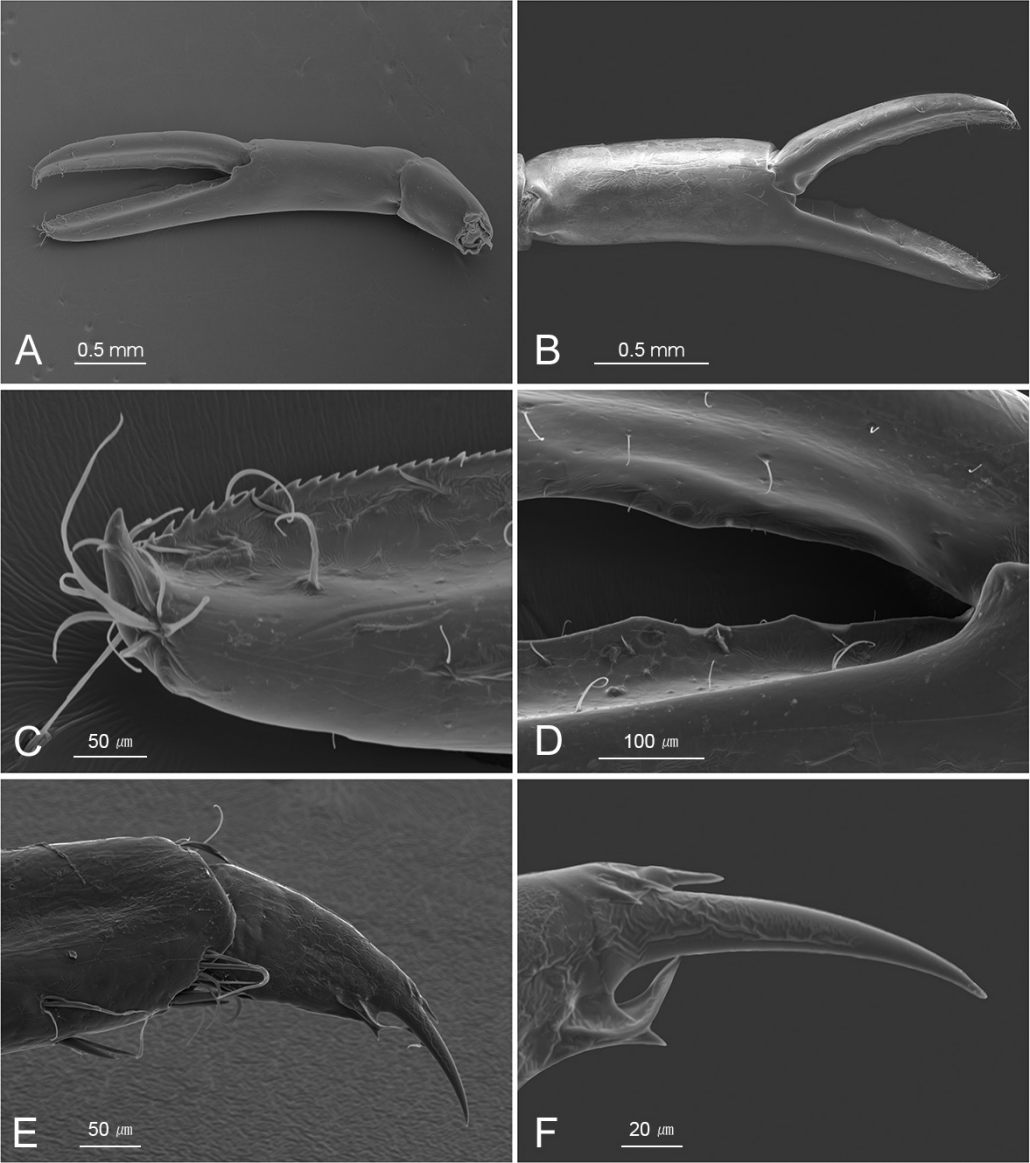
*Cristimenesbrucei* sp. nov., female pocl 2.7 mm (MADBK 120532_002) (**A, C, D**), female pocl 3.34 mm (MADBK 120532_007) (**E, F**), *Cristimenescommensalis* from Vietnam, female pocl 2.0 mm (SNU VI_VI155) (**B**) **A, B** chela and carpus of second pereiopod **C** same, distally serrate margin of fixed finger **D** same, proximally dentate margins of fingers **E** dactylus and distal propodus of third pereiopod **F** same, distal dorsal spinules of dactylar corpus, and unguis.

The new species is morphologically very similar to the other crinoid-associated species in the genus, *C.commensalis* (Fig. 8C). Within the genus, both species share the following characteristics: subspatulate fingers of the first pereiopods; proximally dentate and distally serrate cutting edges of the fingers of the second pereiopods (Fig. 10C, D); and the presence of accessory spinules on the anterior margin of the dactyli of the ambulatory pereiopods. The new species can, however, be easily distinguished from *C.commensalis* by the reduced, blunt supraorbital tooth (Fig. 9A–C) and reduced rostral carinae (vs. well-developed supraorbital tooth (Fig. 9D) and rostral carinae in *C.commensalis*).

*Cristimenesbrucei* sp. nov. can easily be distinguished from the echinoid-associated species *C.cristimanus* (Fig. 8D) and *C.zanzibaricus* by the reduced supraorbital tooth and rostral carinae (vs. extremely developed supraorbital tooth and rostral carinae), the presence of accessory spinules on the anterior margin of the ambulatory dactylus (vs. absent), and a different host affiliation, with the latter two species being associated with echinoids.

The crinoid-associated genera *Araiopontonia* Fujino & Miyake, 1970, *Laomenes* Clark, 1919, and *Unguicaris* Marin & Chan, 2006 are phylogenetically closely related to *Cristimenes*. The new species shares a morphological trait with *Araiopontoniaodontorhyncha* Fujino & Miyake, 1970 in having accessory spinules on the anterior margin of the ambulatory dactylus, but the new species can easily be distinguished from *A.odontorhyncha* by the reduced supraorbital teeth and rostral carinae, the presence of a hepatic tooth on the carapace, and the low and rounded epistome (vs. developed supraorbital tooth and rostral carinae, absence of hepatic tooth, and well developed rounded epistomial horns in *A.odontorhyncha*). All species in the genus *Laomenes* can be distinguished from the new species by having more strongly developed supraorbital teeth and rostral carinae, well developed sharp epistomial horns and simple biunguiculat ambulatory dactylus. The new species is morphologically similar to *U.novaecaledoniae* (Bruce, 1968) among species of the genus *Unguicaris*. The new species shares with *U.novaecaledoniae* similar first chelipeds, proximally dentate but distally serrate cutting edges of the fingers of the second pereiopods, and the presence of well-developed accessory spinules on the anterior margin of the ambulatory dactyli. The new species can, however, be distinguished from *U.novaecaledonia* by the presence of reduced supraorbital teeth (vs. absent).

### Phylogenetic analyses

We obtained fragments of 658, 462, 293, and 655 bp for the COI, 16S, H3, and 18S markers, respectively. Barcode COI regions were calculated for 13 specimens across all four species of *Cristimenes*, with the maximum K2P intraspecific divergence being 0.15%, 1.09% and 1.25% in *Cristimenes*brucei sp. nov., *C.commensalis*, and *C.cristimanus* (Table 2), whilst mean K2P interspecific distances between *C.brucei* sp. nov. and *C.commensalis*, *C.cristimanus* and *C.zanzibaricus* being 18.2%, 13.8%, and 15.1%, respectively (Table 2).

**Table 2.** Kimura 2-Parameter model distances for COI within and among species of *Cristimenes*. N/A - not available.

**Species**	**Maximum distance within species**	**Mean distance between species**			
		**1**	**2**	**3**	**4**
1. *Cristimenesbrucei* sp. nov.	0.15 %				
2. *Cristimenescristimanus*	1.25 %	13.78 %			
3. *Cristimeneszanzibaricus*	N/A	15.10 %	10.95 %		
4. *Cristimenescommensalis*	1.09 %	18.17 %	17.25 %	14.61 %	

**Figure 11.**
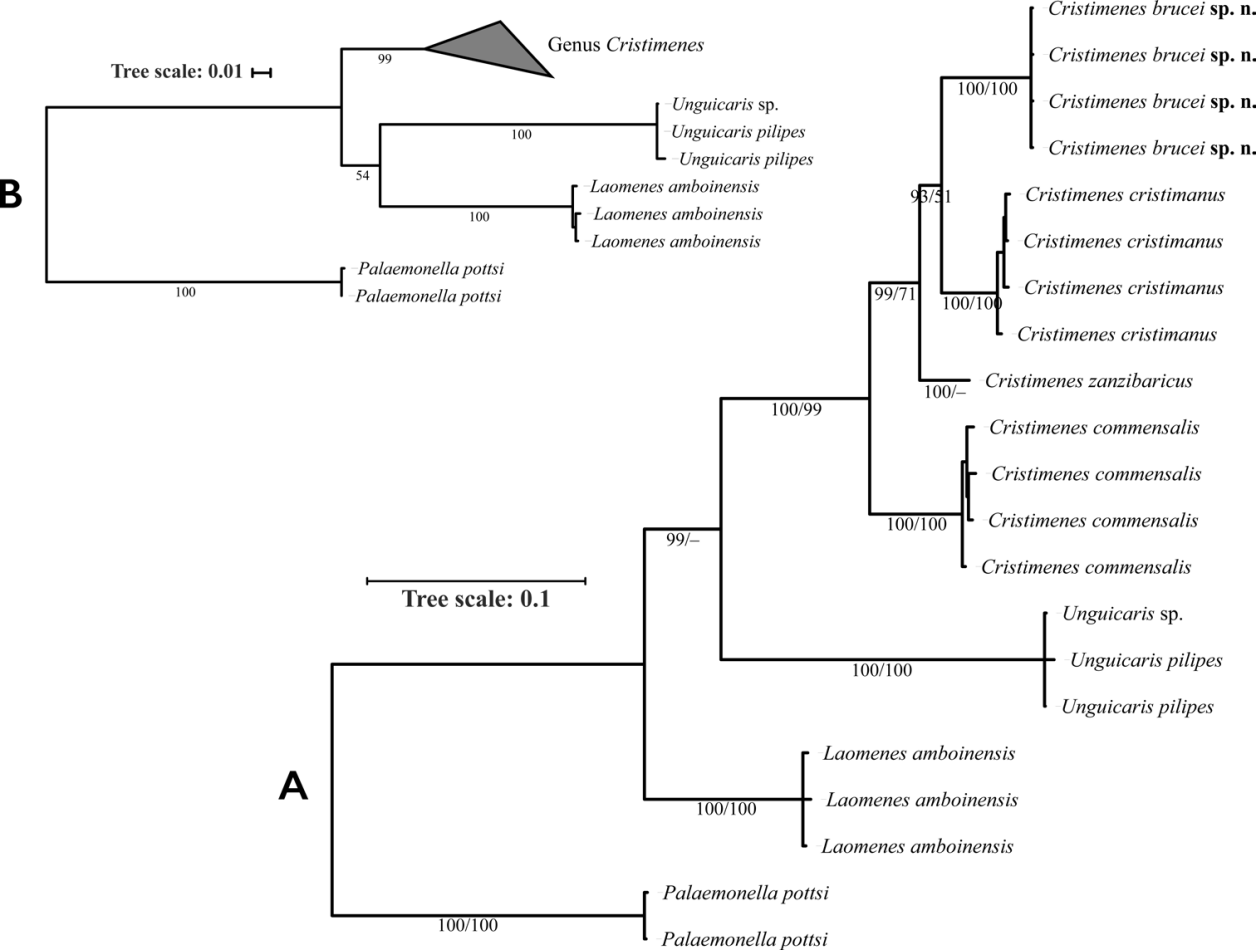
Phylogenetic tree of *Cristimenes* and related crinoid associated genera resolved by Maximum Likelihood (ML) and Bayesian Inference (BI) analysis based on the combined dataset for four genes (COI, 16S, H3, and 18S). BI posterior probabilities and ML bootstrap support (BI/ML) as shown. **A** The topology based on the BI tree **B** Condensed part of ML tree.

Phylogenetic analyses were conducted on 21 specimens of seven species of four genera (Table 1). The combined 2068 bp fragments had 253 parsimony-informative sites for COI, 145 for 16S, 42 for H3, and 102 for 18S. The ML and BI analyses showed the same topology, except for *Laomenes* and *Unguicaris* (Fig. 11). The resulting phylogeny clearly indicates the monophyly of *Cristimenes* with high support values, both in ML and BI analyses. *Cristimenesbrucei* sp. nov. is clearly recovered as a monophyletic species but as a sister group to the echinoid associated *C.cristimanus*, whilst the crinoid associated *C.commensalis* is a sister group to the clade containing *C.brucei* sp. nov., *C.cristimanus*, and *C.zanzibaricus*.

## Acknowledgements

We thank Dr Nguyen Van Tu (ITB, Vietnam) for the invitation and management of fieldwork in Vietnam. We are also grateful to Dr Hyi-Seung Lee (KIOST, Korea) and Dr Wilfredo L. Campos (UPV, Philippines) for the invitation to participate in the Philippine fieldwork. Funding for fieldwork in Vietnam and Philippines was provided through the Marine Biotechnology Program (No. 20170488) funded by the Ministry of Ocean and Fisheries (MOF). JHP is also grateful to Dr Chang-Rae Lee (National Park Research Institute, Korea), Dr Tae Seo Park (NIBR, Korea), and Dr Myung-Hwa Shin (National Marine Biodiversity Institute, Korea) for the invitation to participate in the exploration of the Dadohaesang National Park, the Jeju Special Self-Governing Province, and the East Sea of Korea, respectively. This study is also supported by grant from the National Institute of Biological Resources (No. 201835101), funded by the Ministry of Environment and Marine Biotechnology Program (No. 20170431) funded by the MOF of Korean Government. The authors are grateful to the editor and two reviewers for their valuable comments to this study.

## Supplementary Material

XML Treatment for Cristimenes brucei
